# Doxorubicin-Loaded Lipid Nanoparticles Coated with Calcium Phosphate as a Potential Tool in Human and Canine Osteosarcoma Therapy

**DOI:** 10.3390/pharmaceutics14071362

**Published:** 2022-06-27

**Authors:** Daniela Chirio, Simona Sapino, Giulia Chindamo, Elena Peira, Cristina Vercelli, Chiara Riganti, Maela Manzoli, Graziana Gambino, Giovanni Re, Marina Gallarate

**Affiliations:** 1 Department of Drug Science and Technology, Turin University, Via P. Giuria 9, 10125 Torino, Italy; daniela.chirio@unito.it (D.C.); giulia.chindamo@unito.it (G.C.); elena.peira@unito.it (E.P.); maela.manzoli@unito.it (M.M.); marina.gallarate@unito.it (M.G.); 2 Dipartimento di Scienze Veterinarie, Turin University, Largo P. Braccini, 2, 10095 Grugliasco, Italy; cristina.vercelli@unito.it (C.V.); graziana.gambino@unito.it (G.G.); giovanni.re@unito.it (G.R.); 3 Department of Oncology, Turin University, Regione Gonzole 10, 10043 Orbassano, Italy; chiara.riganti@unito.it

**Keywords:** human osteosarcoma, canine osteosarcoma, microemulsion, nanoparticles, calcium phosphate coating, doxorubicin, uptake, cytotoxicity

## Abstract

Osteosarcoma (OSA) is the most frequently diagnosed primary malignant bone tumor in humans and dogs. In both species, standard chemotherapy can be limited by multidrug resistance of neoplastic cells, which prevents intracellular accumulation of cytotoxic drugs, resulting in chemotherapy failure. In this study, a lipophilic ester of doxorubicin (C12DOXO) was loaded into nanoparticles (NPs) using the “cold microemulsion dilution” method. The resulting NPs were then coated with calcium phosphate (CaP) in two different ways to have calcium or phosphate ions externally exposed on the surface. These systems were characterized by determining mean diameter, zeta potential, and drug entrapment efficiency; afterward, they were tested on human and canine OSA cells to study the role that the coating might play in increasing both drug uptake into tumor cells and cytotoxicity. Mean diameter of the developed NPs was in the 200–300 nm range, zeta potential depended on the coating type, and C12DOXO entrapment efficiency was in the 60–75% range. Results of studies on human and canine OSA cells were very similar and showed an increase in drug uptake and cytotoxicity for CaP-coated NPs, especially when calcium ions were externally exposed. Therefore, applications in both human and veterinary medicine can be planned in the near future.

## 1. Introduction

In recent decades, comparative oncology research has mainly focused on the study of similarities between human and spontaneous canine cancers [1].

Osteosarcoma (OSA) is the most commonly diagnosed primary malignant bone tumor in both humans and dogs. These two species share several similarities in terms of clinical and histological presentation, although the disease is approximately 10 times more common in dogs than in humans [2].

In both species, OSA mainly affects the metaphyseal regions of weight-bearing long bones, is characterized by a highly pleomorphic and heterogeneous microscopic appearance and is divided into several histologic subtypes that may occur simultaneously in the same tumor. The most common subtype in both humans and dogs is the osteoblastic form [3,4].

Classification of OSA is also based on parameters such as cellular pleomorphism, mitotic index, tumor matrix, and degree of necrosis. In most cases, OSA is a high-grade tumor in both species [5].

Depending on the age of the patients at the time of diagnosis, certain differences can be observed, because a bimodal age distribution can be identified with incidence peaks at two different ages. [6]. In dogs, the greatest incidence peak (80% of cases) is found in animals over 7 years of age, while, in humans, the highest frequency of diagnosis occurs in adolescents and young adults (53% of cases under 24 years of age and only 24.3% of cases in individuals over 60 years of age) [7,8].

In both humans and dogs, the gold standard for treatment of OSA is surgery followed by chemotherapy [9] including limb salvage in patients with low-grade tumors, whereas, in patients with high-grade tumors, limb amputation is performed [10].

In humans, the standard adjuvant chemotherapeutic approach involves the use of a combination of methotrexate (MTX), doxorubicin (DOXO), and cisplatin. Administration of additional drugs such as cyclophosphamide has led to conflicting evidence and is not currently a reliable therapeutic alternative [11]. Administration of radiotherapy is a palliative option after postoperative chemotherapy, as OSA has been shown to be highly resistant to radiation [12].

Similar to humans, different approaches (surgery, chemotherapy, or radiotherapy) are required for canine OSA depending on the stadiation of the tumor and the possible presence of metastases. Surgery is considered the gold standard of treatment, alone or in combination with chemotherapy using DOXO [13].

However, the efficacy of DOXO chemotherapy may vary from patient to patient and may be associated with potential side-effects (myelosuppression, cardiotoxicity, and gastrointestinal toxicity). Treatments with carboplatin and cisplatin are considered postoperative chemotherapy that results in a shorter median survival than that achievable with DOXO [14]. Standard chemotherapy can be limited by multidrug resistance (MDR) of neoplastic cells, which is mainly associated with high expression of P-glycoprotein (P-gp). This resistance mechanism prevents the intracellular accumulation of cytotoxic drugs (e.g., DOXO, vinblastine, vincristine, and paclitaxel) in neoplastic cells, resulting in chemotherapy failure [15,16]. The expression of proteins related to resistance and belonging to the ABC superfamily has been found in various canine tumors such as lung and breast tumors, hepatocellular carcinomas, and lymphomas [17].

Considering all these factors, scientific research on OSA is currently aimed at developing new treatment strategies for both human and canine osteosarcomas, where overall survival remains limited [18]. A particular goal pursued is to improve long-term overall survival and reduce adverse reactions; with the use of suitable carriers able to increase the cell drug uptake, a lower dose of antitumor molecules could be administered, thus reducing adverse effects.

The use of nanotechnology has been proposed as a new approach for the treatment of OSA in both veterinary [19] and human [20] settings to avoid multidrug resistance and reduce the negative adverse reactions of most chemotherapeutic agents. In the world of nanomaterials, nanoparticles (NPs) are widely studied for the diagnosis and treatment of OSA due to their peculiar structure, desirable drug entrapment efficiency, and good bioavailability [21,22]. They can be considered as promising tools for the delivery of various chemicals, drugs, small molecules, peptides, nucleic acids, and even vaccines to target sites.

Whilst, in human medicine, several NP-based drugs are currently registered in USA and others are under investigation in clinical trials [23], in veterinary medicine, NP and liposome formulations remain limited to the in vitro setting [24].

The use of molecules with high affinity for bone is another strategy that has been explored to deliver drugs into OSA [25]. In particular, calcium phosphates (CaP) NPs are considered as promising nanocarriers to bone tissues as they were shown to be preferentially accumulated in bone tissues [26,27].

In recent years, only a few attempts have been made to prepare CaP-coated nanostructures suitable as drug delivery systems, such as CaP-coated dioleoyl phosphatidic acid (DOPA) liposomes [28] or CaP nanoshells [29]. More recently, some authors [30] demonstrated that CaP-coated DOPA liposomes were able to strongly interact with *Staphylococcus aureus*, suggesting their use to control biofilm growth.

So far, no one has proposed CaP-coated lipid NPs as drug delivery systems for bone diseases. Given their high versatility, biocompatibility, and kinetic and physical stability compared to liposomes, NPs have been widely applied as nanocarriers for drug delivery [31].

In a previous paper [32], we presented the development and the physicochemical characterization of lipid NPs coated with calcium phosphate (CaP-NPs). The novelty lies in the fact that, for the first time, a layer-by-layer CaP coating was applied to such lipid NPs. Starting from µEs at different compositions, NPs with differently charged surfaces were obtained, serving as attachment points for the coating salts that precipitated layer by layer. Preliminary uptake studies on human osteosarcoma cells (U-2OS) of Sudan Red III-loaded NPs revealed higher cell accumulation of CaP-NPs than uncoated NPs

In this work, DOXO lauroyl ester (C12DOXO), a lipophilic derivative of DOXO, was synthesized and loaded into the previously cited CaP-NPs, the composition of which was further optimized. The aim was to assess whether the developed CaP coating could increase the affinity of C12DOXO-loaded NPs for both human and canine OSA cells.

## 2. Materials and Methods

### 2.1. Chemicals

Trilaurin (purity ≥ 99%), ethyl acetate (EA, purity ≥ 99.5%), propylene glycol (purity ≥ 99.5%), sodium hydroxide (purity ≥ 98%), calcium chloride (purity ≥ 96%), sodium phosphate dibasic dihydrate (purity ≥ 99%), and Sepharose^®^ CL4B were from Sigma Aldrich (St. Louis, MO, USA). Epikuron^®^200 (soybean lecithin, containing 95% phosphatidylcholine) was from Cargill (Minneapolis, MN, USA); Cremophor^®^RH60 (PEG-60 hydrogenated castor oil) was from Acef (Piacenza, Italy); sodium chloride (purity ≥ 99%) and phosphoric acid (≥85% *w*/*w* in H_2_O) were from Alfa Aesar (Haverhill, MA, USA).

#### C12DOXO Synthesis

DOXO lauroyl ester (C12DOXO) was synthesized and characterized as reported in a preview work [33].

### 2.2. Preparation of NPs and C12DOXO-Loaded NPs

Lipid NPs (hereafter referred to as NPs) were prepared using the “cold microemulsion dilution” method [34] that we developed previously. Briefly, an oil-in-water µE was first prepared by mixing 200 µL of a water-saturated EA (s-EA) lipid solution with appropriate amounts of surfactant, cosurfactant, and cosolvent, obtaining the lipid phase, to which 700 µL of EA-saturated water (s-water) was then added dropwise. The mixture was vortexed at room temperature until it became transparent. Trilaurin dissolved in s-EA was used as the lipid, while Epikuron^®^200, Cremophor^®^RH60, and propylene glycol were used as the surfactant, cosurfactant, and cosolvent, respectively. Thereafter, the prepared µE was diluted with 5 mL of water at room temperature to ensure that the organic solvent diffused from the internal phase to the added water, precipitating the NPs. The presence of a charge on the surface of the NP required for the coating process was obtained by exploiting the negative charge of Epikuron^®^200.

The same method was also used to prepare C12DOXO-loaded NPs (C12DOXO NPs). Different amounts of the drug were dissolved in the lipid phase. The compositions of the µEs developed are listed in Table 1.

### 2.3. Purification of NPs

First, 0.5 mL of freshly prepared NPs underwent gel filtration using a stationary crosslinked agarose matrix (Sepharose^®^CL4B) and 0.3 M NaCl mobile phase. Following this process, to remove the excess salts and unentrapped molecules, the resulting opalescent fraction (3 mL) containing the purified NPs was dialyzed overnight at room temperature against ultrapure water using a dialysis bag (MWCO = 14,000 Da). A similar procedure was adopted for C12DOXO NPs.

### 2.4. CaP Coating

CaP coating was performed according to the method developed in a previous study [32]. Briefly, the purified NPs/C12DOXO NPs were diluted 1:2 with ultrapure water, and the coating salts (CaCl_2_ and Na_2_HPO_4_) were added layer by layer to the suspensions. The mixture was magnetically stirred for 20 min after each addition. Over the course of the coating process, pH was monitored and maintained at 8.5 to ensure two negative charges on the phosphate group. The same procedure was followed with C12DOXO NPs.

Two different layer-by-layer sequences (described below) were used to coat the NP formulations to obtain two series of NPs with calcium or phosphate ions externally exposed on the surface (the calcium or phosphate salts were added as a final step during the coating process).

1. NPs/C12DOXO NPs with calcium externally exposed (CaP_Ca_-NPs/CaP_Ca_-C12DOXO NPs) were obtained by adding layer by layer to the purified suspension 300 μL of 42 mM CaCl_2_, then 300 μL of 42 mM Na_2_HPO_4_, and finally 600 μL of 42 mM CaCl_2_
2. NPs/C12DOXO NPs with phosphate externally exposed (CaP_P_-NPs and/CaP_P_-C12DOXO NPs) were obtained by adding layer by layer to the purified suspension 300 μL of 42 mM CaCl_2_, then 300 μL of 42 mM Na_2_HPO_4_, then 300 μL of 42 mM CaCl_2_, and finally 300 μL of 42 mM Na_2_HPO_4_

The resulting suspensions of CaP-NPs were then dialyzed overnight at room temperature against ultrapure water with an MWCO of 14,000 Da to remove the excess ions.

### 2.5. Characterization of NPs

To verify the deposition of CaP on the surface of the NPs, all characterization analyses were performed on both uncoated and coated NPs and on C12DOXO NPs.

#### 2.5.1. Particle Size and Zeta Potential Measurements

Mean diameters of NPs and C12DOXO NPs, as well as of CaP-NPs and CaP-C12DOXO NPs, were measured in triplicate using dynamic light scattering (DLS, Brookhaven Instruments, Holtsville, NY, USA). Zeta potential was measured following adequate dilution of NPs dispersions with ultrapure water using dynamic light scattering (DLS, Brookhaven Instruments, Holtsville, NY, USA) in zeta potential mode, averaging 10 measurements.

#### 2.5.2. C12DOXO Entrapment Efficiency

The entrapment efficiency (%EE) of C12DOXO in the different NPs was evaluated by HPLC, determining the concentration of C12DOXO after and before gel filtration with Sepharose^®^ CL4B. More specifically, the unpurified NP suspension (pre-column) and the fraction containing purified NPs eluted by gel filtration (post-column) were analyzed after appropriate dilution. %EE was determined as the ratio between the amount of drug in the post-column fraction and the amount in the pre-column suspension × 100. HPLC analysis was performed using an LC9 pump (Shimadzu, Tokyo, Japan) with a Teknokroma^®^ ODS column (5 µm, 15 cm × 0.46) and a C-R5A integrator (Shimadzu, Tokyo, Japan); the detector was a fluorimeter with λ_ex_ = 480 nm and λ_em_ = 520 (Shimadzu, Tokyo, Japan). C12DOXO was eluted using a gradient system (0.3% H_3_PO_4_ in water/0.1% H_3_PO_4_ in CH_3_CN) at a flow rate of 1.0 mL/min. The injection volume was 20 µL, and the retention time was 10 min.

A calibration curve with acceptable linearity (*R*^2^ = 0.9997) was built by plotting the peak area as a function of drug concentration in the 1.5–50 µg/mL range. The relative standard deviation (RSD) of intra- and inter-day precision for three concentrations (3, 10, and 20 µg/mL) was below 3% with accuracy ranging from 97.0% to 103.0% HPLC.

#### 2.5.3. FESEM Characterization

Field-emission scanning electron microscopy (FESEM) analyses were carried out using a Tescan S9000G FESEM 3010 microscope working at 30 kV, equipped with a high-brightness Schottky emitter and fitted with energy-dispersive X-ray spectroscopy (EDS) analysis with an Ultim Max Silicon Drift Detector (SDD, Oxford, UK). For analyses, a few drops of naked NPs (blank sample) or CaP_Ca_-NPs were placed on an aluminum stub coated with conductive adhesive and left to dry in vacuum overnight. The as-prepared nonconductive samples were covered by a sputtered nanometric layer (<5 nm) of conductive Cr and then inserted into the chamber using a fully motorized procedure.

#### 2.5.4. In Vitro Release Study

The release of C12DOXO from NPs was estimated in vitro using a multicompartmental rotating cell system consisting of donor and receptor compartments of equal volume (1.5 mL) separated by a membrane with 0.1 μm pores [35]. In separate experiments, C12DOXO in 0.1% Tween^®^80 solution, NP6, and CaP_Ca_-NP6 suspension were used as donor formulations. The 0.1% Tween^®^80 water solution (pH 7.8) was employed as the receiving medium. To achieve sink conditions, at fixed times, the receptor solution was withdrawn, and the compartment was refilled with fresh receiving medium. HPLC was used to determine the concentration of C12DOXO in the receiving medium.

### 2.6. In Vitro Cell Studies

#### 2.6.1. Human Osteosarcoma U-2OS Cells

Human osteosarcoma U-2OS cells (HTB-96, ATCC, Manassas, VA, USA) were cultured in a humidified atmosphere at 37 °C, with 5% CO_2_, in Dulbecco’s modified Eagle’s medium (DMEM) (Invitrogen, Milan, Italy) supplemented with 1% *v*/*v* penicillin–streptomycin (Sigma-Merck, St. Louis, MO, USA) and 10% *v*/*v* fetal bovine serum (Sigma-Merck, St. Louis, MO, USA). U-2OS/DX (DOXO resistant) cells were selected by stepwise selection in medium with increasing concentration of DOXO up to 580 ng/mL DOXO, as reported [36]. Cells were cultured in DMEM containing this DOXO concentration.

#### 2.6.2. Canine Osteosarcoma D17 Cells

Canine osteosarcoma D17 cells (CRL-8468, ATCC, Manassas, VA, USA) were cultured in a 37 °C and 5% CO_2_ atmosphere in the presence of complete medium composed of DMEM, FBS 10%, antibiotic and antimycotic solution 2%, and l-glutamine 2%. All reagents were purchased from Sigma Aldrich (Merck company, St. Louis, MO, USA).

#### 2.6.3. Cytotoxicity Assay

The cytotoxicity assay was performed on both human and canine OSA cells according to the method proposed by Vercelli and coworkers [37]. Briefly, cells were seeded at a concentration of 5 × 10^3^ cells/100 µL in 96-well plates in the presence of complete medium to perform the 3-(4,5-dimethylthiazol-2-yl)-2,5-diphenyltetrazolium bromide (MTT) assay 24, 48, and 72 h after seeding. The plate was divided to obtain the first column without cells (as a blank), the second column with cells but without drug (as a control), and columns 3–12 with different concentrations of NPs. After overnight incubation at 37 °C and 5% CO_2_, the medium was replaced with complete medium containing a reduced amount of FBS (5%) to avoid affecting cell growth. In a preliminary experiment, cells were incubated for 24, 48, and 72 h with increasing concentrations of blank NPs obtained by properly diluting the formulation (1:1000, 1:100, and 1:10 corresponding to 10, 100, and 1000 µg/mL trilaurin, respectively). Afterward, cells were incubated with different concentrations of C12DOXO-loaded NP6 series obtained by diluting the formulation so as to incubate the cells with 5, 2.5, 1, 0.5, and 0.1 μg/mL of C12DOXO. The plate was divided to obtain the first column without cells (as a blank), the second column with cells but without drug (as a control), and the remaining columns with different NPs concentrations.

At the end of each time point, 20 µL of MTT solution (5 mg/mL in PBS) was added to each well. After 4 h of incubation at 37 °C, the content of each well was removed, and 150 µL of DMSO was added to lyse the precipitates of formazan salt. Plates were maintained for 10 min on a shaking plate at room temperature, and the optical density of the wells was determined using a plate reader (Poverwave x; Bio-Tek Instruments Inc., Winooski, VT, USA) at a wavelength (λ) of 570 nm. The results were expressed as the percentage of viable cells, comparing the results obtained per each well of treated cells with the mean value of control cells.

#### 2.6.4. C12DOXO Cellular Uptake

Cells were seeded in 24-well plates and incubated for 3, 6, and 24 h with NPs loaded with 0.1, 0.5, 1.25, 2.5, or 5 μg/μL C12DOXO. At the end of the incubation period, the culture medium was aspirated; then, the cells were washed twice with PBS, detached with 0.50 μL of trypsin, and suspended in 250 μL of PBS. An aliquot of 50 μL was sonicated and used for protein quantification with the BCA-1 kit (Sigma-Merck). The remaining cell suspension was transferred to a 96-well plate and used to read the intracellular fluorescence of C12DOXO as an index of drug uptake using a Synergy HTX multiplate reader. The excitation and emission wavelengths were 596 and 615 nm, respectively. Fluorescence units were converted to nmol of C12DOXO on the basis of a calibration curve generated using free C12DOXO solutions at the following concentrations: 1, 10, 100, 250, and 500 nmol/mL. The results were expressed as nmol/mg cellular proteins.

#### 2.6.5. Statistical Analysis

The results were analyzed using one-way variance analysis (ANOVA) and the Tukey test, using the GraphPad Prism software (v 6.01). A *p*-value < 0.05 was noted as significant. All data were expressed as means ± SD.

## 3. Results and Discussion

DOXO is one of the most commonly used drugs in the treatment of OSA. However, its efficacy against this tumor is limited because cancer cells can develop mechanisms of multidrug resistance (MDR) due to transmembrane drug efflux pumps that actively reduce the intracellular drug to levels below the effective cytotoxic concentration. Consequently, to be effective as an antitumor agent, higher doses of DOXO are required, which increases the risk of toxicity to normal cells, as well as systemic toxicity to most major organs, particularly cardiotoxicity [38] and bone marrow suppression. Therefore, the clinical use of DOXO is often limited. One of the effective approaches to overcome MDR [39,40,41] is to use NPs as drug delivery systems to increase drug accumulation in drug-resistant cancer cells. The expected mechanism was that DOXO-loaded NPs could enter the cells via an endocytic pathway, thus bypassing the P-gp-dependent efflux, leading to increased intracellular drug concentration and drug cytotoxicity [41].

In a previous study [32], we presented a new method for coating lipid NPs with CaP to increase their affinity for OSA cells. NPs with positive or negative surface charge were prepared to use both charge typologies as attachment points thanks to ionic interactions with the coating salts. Since negatively charged NPs were the most suitable systems for coating with CaP and showed the highest cellular uptake of Sudan Red III, which was used as a model molecule, in the present work, NP0 was chosen as the starting point for the preparation of NPs coated with CaP with different final charge.

In this work, a lipophilic ester (C12DOXO) was synthesized to load DOXO into lipid NPs. Starting from the best formulation resulting from our previous work (NP0 in [32]), some samples were loaded with different amounts of C12DOXO and coated with two different CaP coating types.

Thus, we obtained two different NP series, one with phosphate ions externally exposed on the surface (phosphate as the last layer in the coating process, CaP_P_-C12DOXO NPs) and one with calcium ions externally exposed (calcium salts as the last addition during the coating process, CaP_Ca_-C12DOXO NPs). The influence of coating type on OSA cellular uptake was investigated using both human and canine cells.

### 3.1. Characterization of NPs

#### 3.1.1. Particle Size, Zeta Potential, and C12DOXO Entrapment Efficiency Measurements

In Table 2, the characterization data of NPs are reported.

All NPs showed mean diameters in the 200–300 nm range with values that increased with the amount of entrapped C12DOXO. A slight increase in mean diameter was also observed in CaP-coated NPs compared to uncoated, probably due to the deposition of salts on the surface of NPs.

The negative zeta potential of NP0 was due to the phosphate groups of Epikuron^®^200, as mentioned above, which also persisted in the presence of C12DOXO, although it became slightly less negative with increasing amounts of C12DOXO. Unexpectedly, Zeta potential reached a strongly positive value when the amount of loaded C12DOXO was 6 mg; we hypothesize that a not insignificant amount of the drug might have been adsorbed on the NP surface exposing protonated amino groups outward.

To check the coating formation, zeta potential was monitored during the coating process. As already observed in our previous work [32], there was a marked change in zeta potential values after each addition of salt (data not reported). The alternation of zeta potential values could indicate the layer-by-layer deposition of the added ions around the NPs.

In each NP series, a significant increase in zeta potential was observed when CaCl_2_ solution was added as a final step during the coating process; although positive values were never reached, we can assume that predominantly calcium ions were adsorbed onto the surface of the NPs. On the contrary, when Na_2_HPO_4_ solution was added as a final step during the coating process, strongly negative values of the zeta potential were observed, indicating that phosphate ions were adsorbed on the surface of the NPs.

The presence of C12DOXO only slightly changed the zeta potential values in both CaP-NPs series.

The low absolute value of the zeta potential, particularly for CaP_Ca_-NP6, could represent a problem regarding aggregation phenomena. In any case, our idea would be to freeze-dry samples, to increase their stability over time. In our previous manuscript [32], we freeze-dried our NPs and obtained satisfactory results in dispersion. In any case, the peculiarity of the CaP coating could also allow us to modulate the layering of calcium ions and phosphate ions so as to optimize the zeta potential value.

Table 2 presents the %EE of C12DOXO in uncoated and coated NPs. The %EE of the drug in uncoated NPs was in the 80–90% range but fell by about 15–20% after CaP coating, probably because of the agitation and dialysis processes.

#### 3.1.2. FESEM Characterization

**Figure 1:**
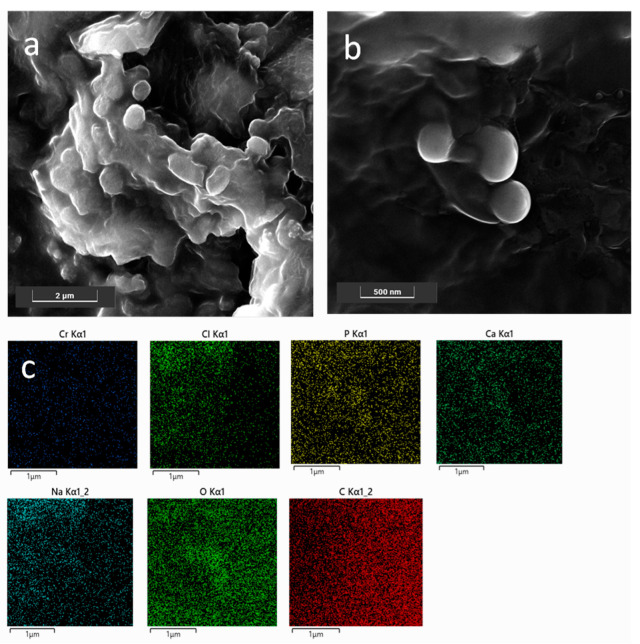
FESEM representative images of bare NPs (**a**) and CaP_Ca_-NPs (**b**); EDS maps of the region shown in (**b**) for Cr, Cl, P, Ca, Na, O, and C (**c**). Images were collected at 10 kV with the standard SE detector and 15 kV with the in-beam SE detector. Instrumental magnification: 30,000× and 10,000×.

FESEM analyses were carried out in order (i) to obtain information on both morphology and size of the NPs, and (ii) to check the presence of the CaP coating on the SLN NPs; the latter point allowed further validation of the preparation procedure. As shown in Figure 1a, bare NP0s with a globular and slightly platelet shape, with size ranging approximately between 200 nm and 400 nm, were observed in agreement with the DLS results. Moreover, the NP0s appeared as embedded in a matrix formed upon drying of the solution under vacuum. Conversely, CaP_Ca_-NP0s had an almost spherical shape and size slightly lower than that of the bare NP0s, as shown in Figure 1b. This was probably due to the presence of the CaP coating, which acted as a stabilizing agent of the NPs, by avoiding coalescence during the drying step under vacuum carried out to prepare the sample for the measurements. The formation of the CaP coating was put in evidence by the EDS maps (Figure 1c) highlighting the presence of Ca, O, and P elements around the coated NPs.

### 3.2. In Vitro Release Study

**Figure 2:**
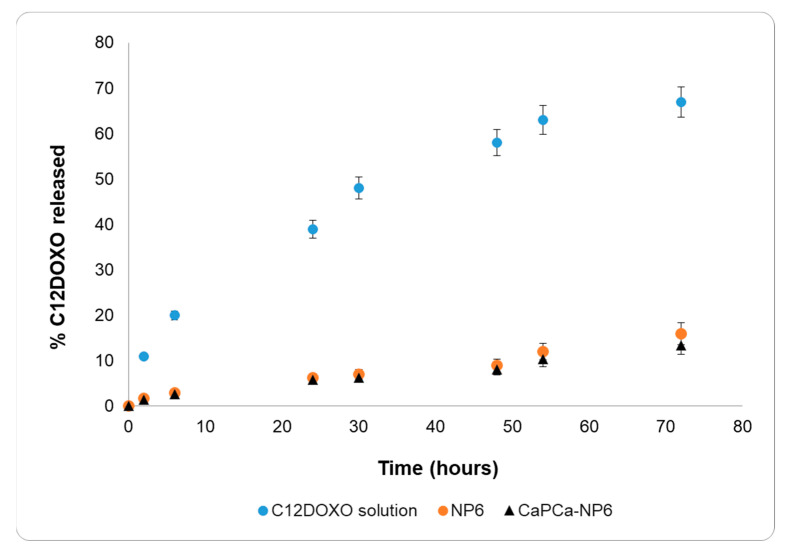
C12DOXO release profiles from 0.1% *w*/*w* Tween^®^80 aqueous solution, NP6, and CaP_Ca_-NP6.

The C12DOXO release study was performed on NP6 and CaP_Ca_-NP6 using an aqueous C12DOXO solution in 0.1% *w*/*w* Tween^®^80 as a reference (Figure 2).

The release data of the C12DOXO solution and both NP6 and CaP_Ca_-NP6 were very different. The release of C12DOXO from the solution was very rapid, reaching 70% within 3 days and showing a release profile typical of solutions. On the contrary, C12DOXO was released more slowly from NP6 and CaP_Ca_-NP6, reaching only 15% within 3 days, showing pseudo-zero-order kinetics in the time interval considered. No burst effect was observed with NP6 and CaP_Ca_-NP6, actually suggesting that all the loaded C12DOXO was entrapped into the lipid matrix. Indeed, a burst-effect release pattern generally occurs in nanostructured systems with large amounts of non-entrapped or surface-bound drug.

According to these results, the diffusion of C12DOXO in the lipid matrix is the most important rate-determining step of the release, and the difference in the release from the solution and NPs can, thus, be attributed to the entrapment of C12DOXO in the lipid core.

The results of in vitro release, thus, confirm the entrapment of C12DOXO in NPs.

### 3.3. In Vitro Cell Studies

#### 3.3.1. Human Osteosarcoma U-2OS Cells

**Figure 3:**
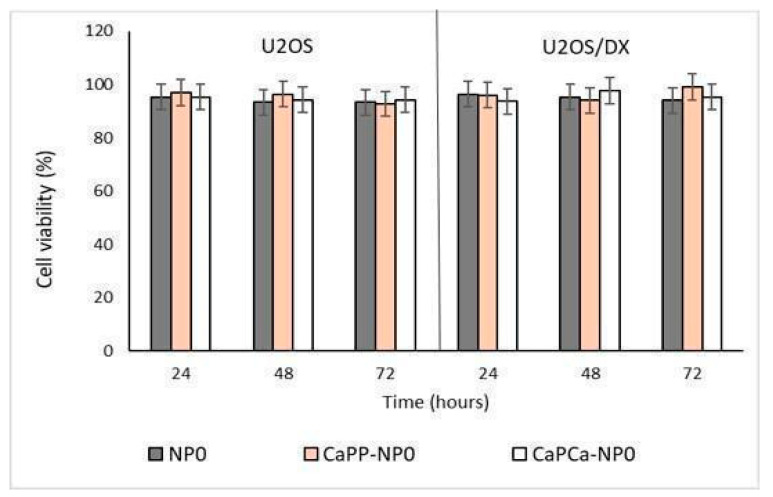
U-2OS and U-2OS/DX cells were incubated with NP0, CaP_P_-NP0, and CaP_Ca_-NP0, diluted 1:10 (1000 µg/mL trilaurin) in the cell culture medium, for 24, 48 and 72 h. Cell viability was measured using the MTT assay. Results are means ± SD (*n* = 3).

In a preliminary experiment, we incubated DOXO-sensitive U-2OS and DOXO-resistant U-2OS/DX cells with increasing concentrations of unloaded NPs, reaching a final dilution of 1:1000, 1:100, and 1:10 (corresponding to 10, 100, and 1000 µg/mL trilaurin, respectively) for 24, 48 and 72 h to evaluate their eventual intrinsic cytotoxicity. The different NP formulations did not reduce cell viability by more than 20% in sensitive U-2OS or resistant U-2OS/DX cells, at each concentration and each timepoint (Supplementary Figures S1a,b and Figure 3).

**Figure 4:**
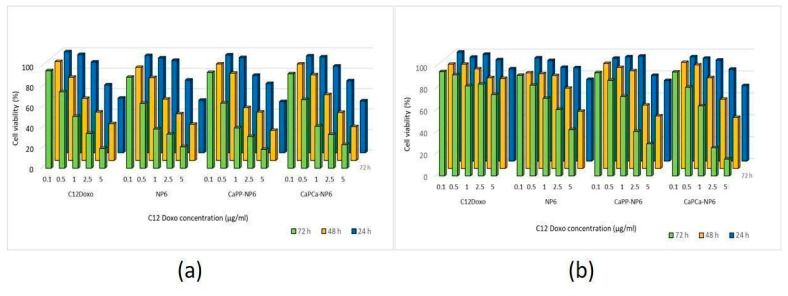
U-2OS (**a**) and U-2OS/DX (**b**) cells were incubated with NP6, CaP_P_-NP6, and CaP_Ca_-NP6, carrying 0.1, 0.5, 1, 2.5, or 5 µg/mL C12DOXO for 24, 48, and 72 h. Cell viability was measured using the MTT assay. Results are means ± SD (*n* = 3). U-2OS, 24 h: *p* < 0.05 for CaP_P_-NP6 (1 µg/mL); *p* < 0.001 for C12DOXO, NP6, CaP_P_-NP6, and CaP_Ca_-NP6 (2.5 and 5 µg/mL). U-2OS, 48 h: *p* < 0.05 for C12DOXO, NP6, CaP_P_-NP6, and CaP_Ca_-NP6 (all concentrations); *p* < 0.001 for C12DOXO, NP6, CaP_P_-NP6, and CaP_Ca_-NP6 (all concentrations). U-2OS, 72 h: *p* < 0.01 for C12DOXO (0.5 µg/mL); *p* < 0.001 for C12DOXO, NP6, CaP_P_-NP6, and CaP_Ca_-NP6 (all concentrations). U-2OS/DX, 24 h: *p* < 0.05 for NP6 (5 µg/mL); *p* < 0.01 for NP6, CaP_P_-NP6, and CaP_Ca_-NP6 (5 µg/mL). U-2OS/DX, 48 h: *p* < 0.05 for NP6 (2.5 µg/mL) and CaP_Ca_-NP6 1 µg/mL); *p* < 0.001 for NP6 (5 µg/mL), CaP_P_-NP6, and CaP_Ca_-NP6 (2.5 and 5 µg/mL). U-2OS/DX, 72 h: *p* < 0.01 for NP6, CaP_P_-NP6, and CaP_Ca_-NP6 (1 µg/mL); *p* < 0.001 for NP6, CaP_P_-NP6, and CaP_Ca_-NP6 (2.5 and 5 µg/mL).

After verifying the lack of cytotoxicity of each NP formulation diluted 1:10 (1000 µg/mL trilaurin, corresponding to the C12DOXO concentration of 5 µg/mL), we next compared the cytotoxic potential on U-2OS cells of NP6, CaP_P_-NP6, and CaP_Ca_-NP6 loaded with C12DOXO at different concentrations, ranging from 0.1 to 5 µg/mL. Compared with the free drug, we did not detect any significant advantage of NP6, CaP_P_-NP6, and CaP_Ca_-NP6 carrying C12DOXO at each timepoint (Figure 4a).

As expected, C12DOXO was not significantly toxic in U-2OS/DX cells, even at the highest concentration (5 µg/mL) and at the highest timepoint. After 24 h, the NP formulations did not show superior cytotoxicity than C12DOXO, while, after 48 h, the viability of U-2OS/DX cells was significantly lower when C12DOXO-loaded NP6, CaP_P_-NP6, and CaP_Ca_-NP6 were used at 2.5 and 5 µg/mL drug concentration. The reduction in cell viability was even more pronounced after 72 h. Comparing the different formulations, CaP_Ca_-NP6 represented the most cytotoxic NPs, followed by CaP_P_-NP6 and NP6 (Figure 4b).

**Figure 5:**
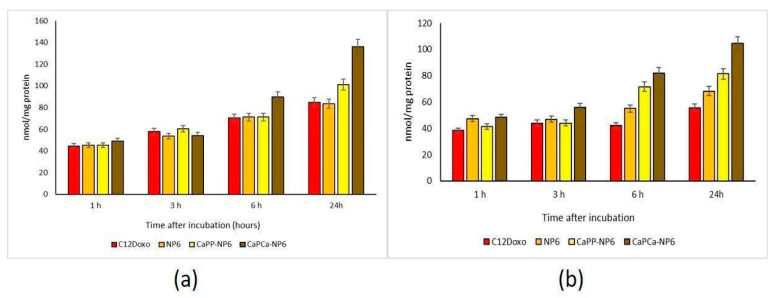
U-2OS (**a**) and U-2OS/DX (**b**) cells were incubated with 5 µg/mL C12 DOXO or with NP6, CaP_P_-NP6, and CaP_Ca_-NP6, carrying 5 µg/mL C12DOXO, for 1, 3, 6, and 24 h. Intracellular retention of DOXO was measured spectrofluorimetrically. Results are means + SD (*n* = 3). U-2OS, 6 h: *p* < 0.01 for C12DOXO, NP6, and CaP_P_-NP6; *p* < 0.001 for CaP_Ca_-NP6. U-2OS, 6 h: *p* < 0.001 for C12DOXO, NP6, CaP_P_-NP6, and CaP_Ca_-NP6 (all parameters compared to 1 h). CaP_P_-NP6 vs. C12DOXO, NP6, and CaP_P_-NP6: *p* < 0.01 at 6 h; *p* < 0.001 at 24 h. U-2OS/DX, 6 h: *p* < 0.01 for CaP_P_-NP6; *p* < 0.001 for CaP_Ca_-NP6. U-2OS/DX, 24 h: *p* < 0.05 for NP6, *p* < 0.01 for CaP_P_-NP6; *p* < 0.001 for CaP_Ca_-NP6 (all parameters compared to 1 h). For NP6 vs. C12DOXO: *p* < 0.05 at 6 and 24 h. CaP_P_-NP6 and CaP_P_-NP6 vs. C12DOXO and NP6: *p* < 0.001 at 6 h and 24 h.

This differential cytotoxicity of NPs versus the free drug could be attributed to a different rate of uptake. In U-2OS cells, we observed a time-dependent increased accumulation of free C12DOXO. Neither NP6 nor CaP_P_-NP6 showed superior uptake compared to the free drug, except for CaP_P_-NP6 after 24 h. In contrast, CaP_Ca_-NP6 was significantly more retained after 6 and 24 h (Figure 5a).

The most striking differences were observed in the resistant cells, where C12DOXO showed a low accumulation at all timepoints, likely due to the efflux of drug via P-gp that is highly expressed in these cells [42]. Interestingly, the use of all NPs determined a significantly increased retention of DOXO at 6 and 24 h. CaP_Ca_-NP6 caused the highest uptake, followed by CaP_P_-NP6 and NP6 (Figure 5b). These results may explain the differential cytotoxicity among the three types of NPs detected in U-2OS/DX cells. Moreover, they also suggest that NP6, particularly the CaP_Ca_-NP formulation, was able to increase the intracellular delivery of DOXO even in cells highly expressing P-gp, the main efflux transporter of the drug in OSA [36]. Since the highest differences between free C12DOXO and CaP_Ca_-NP were observed at longer timepoints, different kinetics of entry of C12DOXO between free drug and CaP_Ca_-NP formulation can be argued. The latter likely determined a slower but sustained and more prolonged release of the drug, justifying the higher cytotoxicity at long term. Overall, CaP_Ca_-NP can be proposed as an effective tool to increase the drug delivery of DOXO, one of the first-line chemotherapeutic agents used in OSA, in resistant human OSA cells.

#### 3.3.2. Canine Osteosarcoma D17 Cells

**Figure 6:**
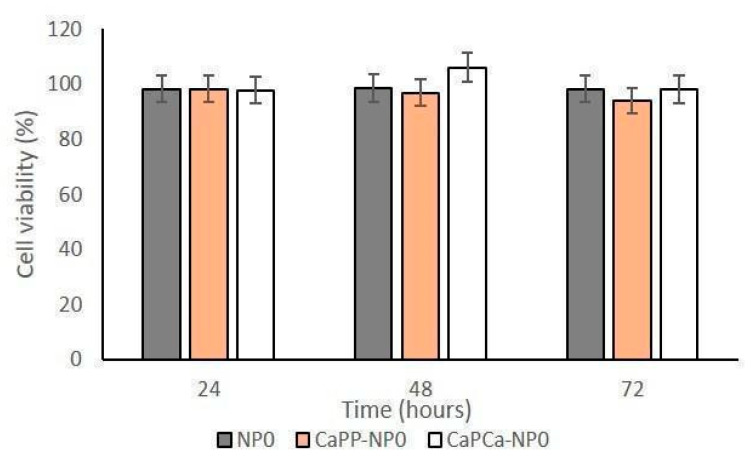
D17 cells were incubated with NP0, CaP_P_-NP0, and Cap_Ca_-NP0, diluted 1:10 (1000 µg/mL trilaurin) in the cell culture medium, for 24, 48, and 72 h. Cell viability was measured using the MTT assay. Results are means + SD (*n* = 0.3).

The proliferation assay dealing with D17 cells in the presence of bare NP formulations demonstrated that no cytotoxic effect was induced (Figure 6 and Supplementary Figure S2), confirming the results obtained with human OSA cells.

**Figure 7:**
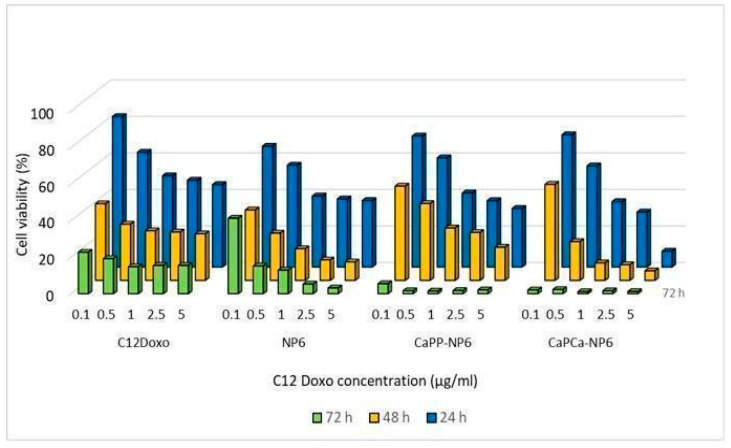
D17 cells were incubated with NP6, CaP_P_-NP6, and CaP_Ca_-NP6, carrying 0.1, 0.5, 1, 2.5, or 5 µg/mL C12DOXO, for 24, 48, and 72 h. Cell viability was measured using the MTT assay. Results are means + SD (*n* = 3).

Therefore, further investigations were planned. NP6, CaP_P_-NP6, and CaP_Ca_-NP6 loaded with C12DOXO were incubated with D17 cells at different concentrations (from 0.1 to 5 µg/mL) to evaluate the proliferation rate at 24, 48, and 72 h after the administration of loaded NP formulations. D17 demonstrated a time- and concentration-dependent decreasing proliferation rate, more evident with CaP_P_-NP6 and CaP_Ca_-NP6. With both formulations, the rate of viable cells after 72 h was dramatically low, mostly at the highest concentrations (Figure 7).

Comparing the results of D17 cells with those of human cell lines, in this case, the CaP_Ca_-NP6 formulation also showed the most effective cytotoxicity. All NP formulations demonstrated a higher efficacy compared to free C12DOXO, considering all tested concentration and for all timepoints.

**Figure 8:**
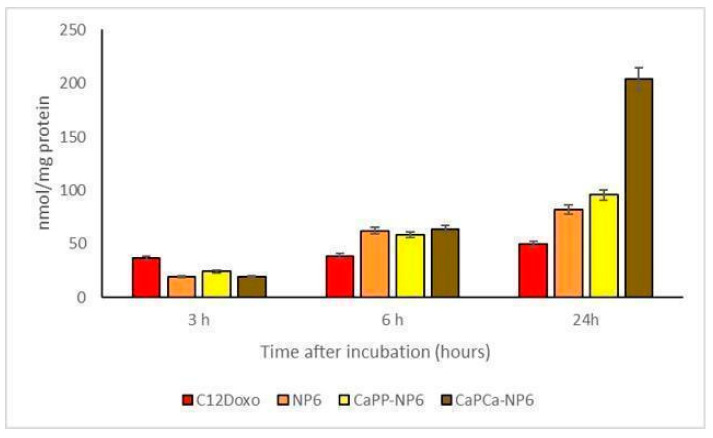
D17 cells were incubated with 5 µg/mL C12DOXO or with NP6, CaP_P_-NP6, and CaP_Ca_-NP6, carrying 5 µg/mL C12DOXO, for 1, 3, 6, and 24 h. Intracellular retention of DOXO was measured spectrofluorimetrically. Results are means ± SD (*n* = 3).

Considering that data obtained by the experiments aimed to evaluate the uptake of the different formulations, different biological effects can be appreciated. For these experimental sessions, different concentrations (ranging from 0.1 to 5 µg/mL) of NP6, CaP_P_-NP6, and CaP_Ca_-NP6 loaded with C12DOXO and C12DOXO alone were tested on D17 cells (Supplementary Figure S3). An increasing uptake of all NP formulations was appreciated if compared to free C12DOXO. This permitted supposing that the NP formulation is able to enhance the uptake of the drug. Moreover, the CaP_Ca_-NP6 formulation showed the best uptake compared to the other two NP formulations. These data are in accordance with what was observed in the cytotoxicity assay, with a better uptake corresponding to a better effect to induce the death of neoplastic cells. Considering only the 5 µg/mL concentration (Figure 8), the increasing trend of uptake in D17 cells was similar to that observed in U-2OS and U-2OS/DX human cell lines, in which a progressive and time-dependent accumulation of NPs was appreciable t the different timepoints, indicating that a longer incubation corresponds to a better uptake.

To the best of the authors’ knowledge, this is the first time that NP formulations presenting a positive or negative charge on the surface have been tested on D17 cells. According to the recent literature, gold NPs have been tested on D17 showing that DOXO complexed with these NPs is able to induce a more potent cytotoxic effect when compared to free DOXO [16]. These results are in accordance with those obtained by the present investigation in D17 cells. Moreover, comparing the concentrations of NP formulations and DOXO, it seems that, using the NP formulations tested in our paper, the final concentration to induce cytotoxicity is lower than that used in experiments with gold NPs.

Overall, the effects of NP6, CaP_P_-NP6, and CaP_Ca_-NP6 loaded with C12DOXO in terms of cytotoxicity and C12DOXO uptake were comparable between human and canine OSA cells. In both cell species, NPs were superior to the free drug, and CaP_Ca_-NP6 was the most effective formulation, confirming their internalization despite their size (200–300 nm). Furthermore, according to literature [43], cell uptake of NPs depends on many parameters, such as size, shape, hydrophobicity, surface charge, and functionality. Probably, cell uptake of the developed NPs could be influenced by their coating (as resulted by C12DOXO uptake study) and could happen via endocytosis. Once inside the tumor cell, they could be degraded by enzymes such as lysosomal hydrolases.

This aspect strengthens the results obtained, indicating no species specificity. Accordingly, CaP_Ca_-NP6 loaded with C12DOXO could be an important tool for the treatment of human OSA refractory to first-line treatment and canine OSA, characterized by a very poor prognosis. Therefore, applications in both human and veterinary medicine can be planned in the near future.

## 4. Conclusions

On the basis of our previous published data [32] indicating the key role that the CaP coating can play in NPs to deliver the entrapped molecule to bone cells, the main objective of this work was to investigate the influence of the type of coating on drug uptake and cytotoxicity. C12DOXO-loaded NPs, prepared using “cold microemulsion dilution” technology, were coated layer by layer with CaP salts in such a way as to have calcium or phosphate externally exposed on the surface.

In vitro cell studies on C12DOXO-loaded NPs evidenced that CaP-coated NPs delivered more drug to the cells within 24 h than the uncoated NPs, showing a key role of the coating in enhancing the delivery of the entrapped molecule to both human and canine OSA cells. In particular, in both cell types, an interesting result was evidenced; the external exposure of calcium significantly increased the C12DOXO uptake and, consequently, the cytotoxic effect. This effect, more evident in the canine cell line, was maintained in human U-2OS/DX, and this result could be considered as a good starting point to overcome the chemoresistance. Indeed, it is noteworthy that CaP_P_-NP6 and, particularly, CaP_Ca_-NP6 loaded with C12DOXO were also cytotoxic against drug-resistant U-2OS/DX cells, whereas the free drug was not. Since our results suggest that the drug was more taken up by the cells, we hypothesize that the CaP coating helps intracellular drug delivery, subtracting the drug from the efflux of P-gp. This observation is further motivation to extensively test, in the near future, C12DOXO-loaded CaP_Ca_-NPs in OSA cells for their association with other molecules able to inhibit the expression of P-gp. This approach may be proposed as a novel strategy to treat multidrug-resistant human and canine OSA.

## Figures and Tables

**Table 1:** Composition of microemulsions and nanoparticles.

		Ingredients (mg)	µE0/ NP0	µE2/ NP2	µE4/ NP4	µE6/ NP6
**Nanoparticles**	**Microemulsions**	Trilaurin	60	60	60	60
		s-EA	200	200	200	200
		Epikuron^®^200	170	170	170	170
		Cremophor^®^RH6	50	35	35	35
		Propylene glycol	100	90	90	90
		s-Water	700	700	700	700
		C12DOXO	-	2	4	6
		Dilution water (mL)	5	5	5	5

**Table 2:** Mean diameter, Zeta potential, and drug entrapment efficiency of different nanoparticles.

	Mean Diameter (nm) PDI	Zeta Potential (mV)	C12DOXO %EE
NP0	209.7 ± 2.8 0.128	−23.5 ± 1.4	-
CaP_Ca_-NP0	232.2 ± 4.0 0.145	−1.6 ± 0.2	-
CaP_P_-NP0	257.5 ± 2.8 0.165	−19.6 ± 3.7	-
NP2	253.4 ± 3.4 0.130	−21.2 ± 1.0	83.2 ± 2.4
CaP_Ca_-NP2	290.2 ± 3.5 0.147	−6.8 ± 0.8	70.0 ± 3.2
CaP_P_-NP2	293.8 ± 4.2 0.176	−18.7 ± 3.1	61.2 ± 4.1
NP4	268.5 ± 2.1 0.153	−14.9 ± 2.0	84.7 ± 3.8
CaP_Ca_-NP4	285.2 ± 2.6 0.188	−4.2 ± 1.0	73.0 ± 2.5
CaP_P_-NP4	311.2 ± 1.6 0.195	−13.5 ± 3.9	62.6 ± 2.4
NP6	282.7 ± 3.2 0.154	+12.7 ± 1.5	87.2 ± 2.9
CaP_Ca_-NP6	311.8 ± 6.3 0.163	+1.6 ± 2.0	74.2 ± 2.4
CaP_P_-NP6	322.7 ± 8.5 0.186	−3.9 ± 0.9	71.5 ± 3.3

## Data Availability

Not applicable.

## Supplementary Materials

The following supporting information can be downloaded at https://www.mdpi.com/article/10.3390/pharmaceutics14071362/s1: Figure S1: U-2OS (a) and U-2OS/DX (b) cells were incubated with NP0, CaP_P_-NP0, and Cap_Ca_-NP0, diluted 1:10, 1:100, and 1:1000 in the cell culture medium, for 24, 48, and 72 h. Cell viability was measured using the MTT assay. Results are means + SD (*n* = 3); Figure S2: D17 cells were incubated with NP0, CaP_P_-NP0, and Cap_Ca_-NP0, diluted 1:10, 1:100, and 1:1000 in the cell culture medium, for 24, 48, and 72 h. Cell viability was measured using the MTT assay. Results are means + SD (*n* = 3); Figure S3: D17 cells were incubated with 0.1, 0.5, 1, 2.5, or 5 µg/mL C12DOXO or with NP6, CaP_P_-NP6, and Cap_Ca_-NP6, carrying 5 µg/mL C12 DOXO, for 1, 3, 6, and 24 h. Intracellular retention of DOXO was measured spectrofluorimetrically. Results are means + SD (*n* = 3).
