# Supplementary material for: Doxorubicin-Loaded Lipid Nanoparticles Coated with Calcium Phosphate as a Potential Tool in Human and Canine Osteosarcoma Therapy
Source: Pharmaceutics. 2022 Jun 27;14(7):1362. doi: 10.3390/pharmaceutics14071362 (PMC9322757; doi:10.3390/pharmaceutics14071362)
Supplement: Supplementary file 1 [file pharmaceutics-14-01362-s001.zip › pharmaceutics-1762594-supplementary.pdf]

Supplementary file

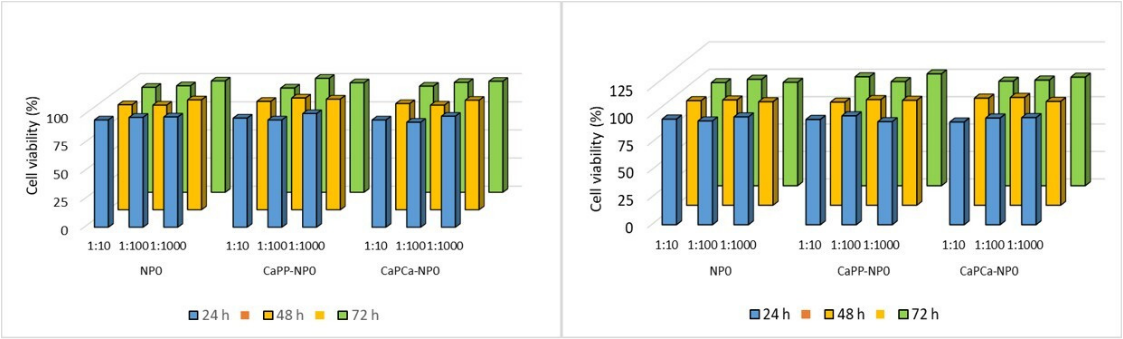

(a) (b)

Figure S1. U-2OS (a) and U-2OS/DX (b) cells were incubated with NP0, CaPP-NP0 and CapCa-NP0, diluted 1:10, 1:100 and 1:1000 in the cell culture medium, for 24, 48 and 72 h. Cell viability was measured by the MTT assay. Results are means ± SD (n=3).

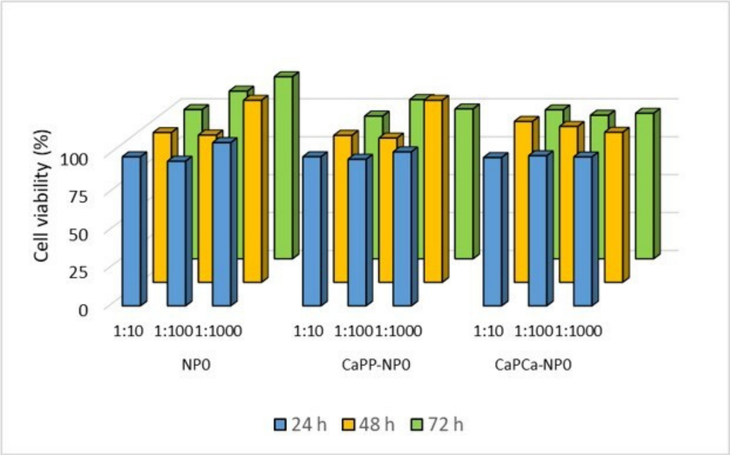

Figure S2. D17 cells were incubated with NP0, CaPP-NP0 and CapCa-NP0, diluted 1:10, 1:100 and 1:1000 in the cell culture medium, for 24, 48 and 72 h. Cell viability was measured by the MTT assay. Results are means ± SD (n=3).

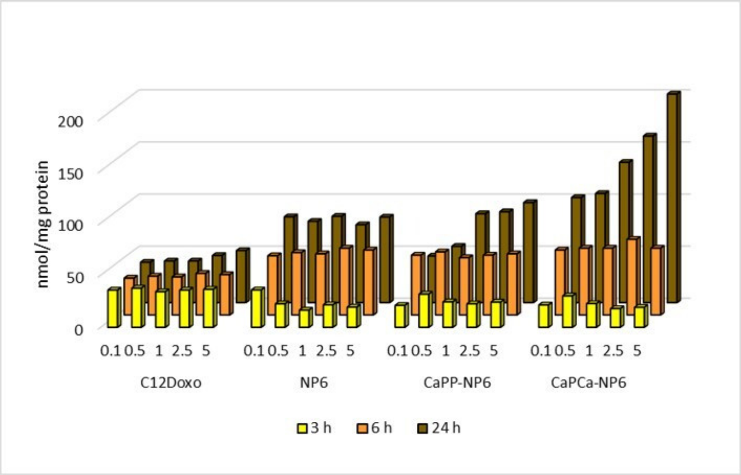

Figure S3. D17 cells were incubated with 0.1, 0.5, 1, 2.5 and 5 µg/mL C12DOXO or with NP6, CaP_P-NP6 and Cap_{Ca}-NP6, carrying 5 µg/mL C12 DOXO, for 1, 3, 6 and 24 h. Intracellular retention of DOXO was measured spectrofluorimetrically. Results are means ± SD (n=3).
